# Changes in Medical Journals

**Published:** 1859-07

**Authors:** 


CHANGES IN MEDICAL JOURNALS.—Drs. Davis and Byford have ceased their connection with the editorship of the *Chicago Medical Journal*, and Dr. Brainard has become their successor.

On account of the removal to New York of Dr. Austin Flint, Jr., the editor of the *Buffalo Medical Journal*, this periodical has been issued under the title of the "*New York Monthly Review of Medical and Surgical Science, and Buffalo Medical Journal.*" It will appear simultaneously in both cities.

The following journals have ceased to exist:—The *Philadelphia Medical and Surgical Journal*, the *Louisville Medical Gazette*, and the *Montreal Medical Chronicle.*

